# Complete chloroplast genome sequence of *Dendranthema zawadskii* Herbich

**DOI:** 10.1080/23802359.2021.1942261

**Published:** 2021-06-28

**Authors:** Sun Hongmei, He Wenrui, Hou Dianyun, Xiuwei Yang

**Affiliations:** a Academy for Advanced Interdisciplinary Studies, Peking University, Beijing, China; b Infinitus (China) Co. Ltd., Jiangmen, China; c Pharmacy College, Chengdu University of Traditional Chinese Medicine, Chengdu, China; d Agricultural College, Henan University of Science and Technology, Luoyang, China; e State Key Laboratory of Natural and Biomimetic Drugs, Department of Natural Medicine, School of Pharmaceutical Science, Peking University, Beijing, China

**Keywords:** *Dendranthema zawadskii* Herbich, chloroplast genome, phylogeny analysis

## Abstract

*Dendranthema zawadskii* Herbich is one of 17 species of *Dendranthema* in China, and it is often used as an ornamental plant. The chloroplast genome size of *Dendranthema zawadskii* Herbich is 150,995 bp, including a large single-copy region (82,771 bp), a small single-copy region (18,308 bp), and a pair of inverted repeats regions (24,958 bp). Total 112 genes were annotated, including 79 protein-coding genes, four ribosomal RNA genes, and 29 transfer RNA genes. The phylogenetic position of *Dendranthema zawadskii* Herbich is close to *Dendranthema indicum*.

*Dendranthema* (DC.) Des Moul. is a genus of Asteraceae, which includes 30 species, most of them are used for ornamental or medicinal purpose. It is mainly distributed in China, Japan, North Korea, and Russia (Song et al. 2018). There are 17 species in China, including *Dendranthema morifolium* Ramat. Tzvel, *Dendranthema indicum* (L.) Des Moul, *Dendranthema lavandulifolium*, *Dendranthema zawadskii* Herbich (Herb.) Tzvel., etc. In addition to ornamental value, most *Dendranthema* genera also have a history and tradition of medicinal use, tea use, and edible use. *D. morifolium* Ramat. Tzvel and *D. indicum* (L.) Des Moul are included in the Chinese Pharmacopeia (2015 edition). The medicinal parts of Chrysanthemum and wild Chrysanthemum are in dry flower heads, and the harvesting time and processing technology are the same in addition to differences in efficacy (Kim 2003). A typical higher plant genome consists of three parts: the nuclear genome, chloroplast genome, and the mitochondrial genome (Hu et al. 2019; Xu et al. 2017). Chloroplasts, the organelles of most green plants, are unswervingly related to the photosynthesis of plants. The function of the genome is also inseparable from photosynthesis. It is generally believed that the chloroplasts are originated from the endophytic symbiont by the cyanobacteria. It is generally patrilineal inheritance in gymnosperms and mostly maternal inheritance in angiosperms. The chloroplast genome of higher plants is normally closed circular DNA, has a highly conserved tetrad structure, with one large single-copy (LSC) region, one small single-copy (SSC) region, and two inverted repeats (IRs) regions with substantially the same length. The chloroplast genome constitutes an ideal data set for evolutionary research and molecular identification, possessing great potential for genetic engineering and traditional Chinese medicine breeding. Here we reported the whole chloroplast genome sequence of *D. zawadskii* Herbich, together with the characterization of its gene annotations and repeat compositions. We compared this chloroplast genome with other species in the genus *Dendranthema* as well as some other related species.

The *D. zawadskii* Herbich planted in Henan University of Science and Technology, Luoyang City, Henan Province (N34°36′11.57″, E112°24′53.56″). The specimen of *D. zawadskii* Herbich is deposited at Asteraceae Specimen Museum of Henan University of Science and Technology (Dianyun Hou, dianyun518@163.com) under voucher number AL2019061803. Fresh *D. zawadskii* Herbich leaves were gathered and covered with tin foil, frozen by liquid nitrogen, and kept in a −80 °C preservation reserve. The CTAB (Qu et al. 1996) method was used to obtain the whole genomic DNA. The ND-2000 spectrometer (Nanodrop Technologies, Wilmington, DE, USA) was used to test the concentration of DNA. The library of 250 bp shotgun was sequenced by the Illumina X ten platform. The raw data was filtered by Skewer-0.2.2(Jiang et al. 2014), and BLAST searches were used to get chloroplast-like (cp) reads from clean-reads in comparison with reference sequences of *Dendranthema boreale* (MN909052.1). ABYSS version 2.0.0 (Simpson et al. 2009) was used for chloroplast genome assembly with cp reads. We downloaded 11 whole chloroplast genome sequences from the NCBI Organelle Genome and Nucleotide Resources database and used all genomes to analyze the phylogenetics. Mafft v7.407 (Nakamura et al. 2018) was used to align genome sequences. MEGA5.2 (Tamura et al. 2011) was used to analyze and plot the phylogenetic tree with neighbor-joining. The chloroplast genome annotation and gene map completion were used CPGAVAS (Liu et al. 2012) platform, and OGDRAW (Lohse et al. 2007) platform, respectively. The accession number in GenBank of *D. zawadskii* Herbich is MW464197.

The size of the *D. zawadskii* Herbich chloroplast genome is 150,995 bp, counting a large single-copy region (82,771 bp, GC:35.6%), a small single-copy region (18,308 bp, GC:30.9%), and a pair of inverted repeats regions (24,958 bp, GC:43.1%). The *D. zawadskii* Herbich chloroplast genome encodes 112 genes, including 79 protein-coding genes, four ribosomal RNA genes, and 29 transfer RNA genes. Seventeen genes—seven tRNA, all four rRNA, and six protein-coding genes—are repeated in the IR regions. The LSC region contains 59 protein-coding and 21 tRNA genes, while the SSC region comprises one tRNA gene and 12 protein-coding genes. In total, there are 15 intron-containing genes, including 10 protein-coding genes and five tRNA genes. Twelve genes (seven protein-coding and five tRNA genes) comprise one intron and three genes (*ycf3*, *clpP*, and *rps12*) comprise two introns.

Phylogenetic analyses were done on an alignment of concatenated nucleotide sequences of all genomes from 12 angiosperm species. The method of neighbor-joining was used to build a phylogenetic tree, and (KX352464.1) was used as the outgroup (Figure 1). The NJ phylogenetic results strongly supported the hypothesis that *D. zawadskii* Herbich and *D. indicum* (JN867592.1) are sister species, and *D. morifolium* (KX522942.1) is more closely related to *D. boreale* (MG913594.1).

**Figure 1.**
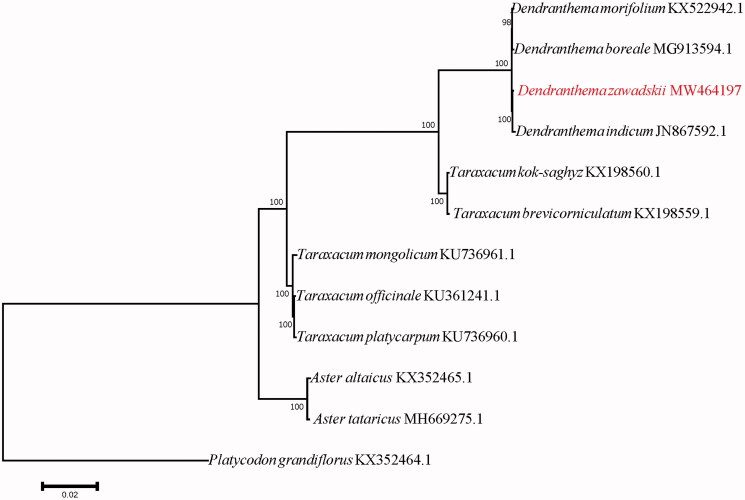
Neighbor-joining phylogenetic tree based on the chloroplast genome of *Dendranthema zawadskii* and other 11 species.

## Data Availability

The genome sequence data that support the findings of this study are openly available in GenBank of NCBI at https://www.ncbi.nlm.nih.gov under the accession No. MW464197. The associated BioProject, SRA, and Bio-Sample numbers are PRJNA727945, SAMN19065990, and SRR14461549, respectively.
